# The dynamic requirements of tryptophan in the broilers at different growing stages under low-protein diets

**DOI:** 10.1016/j.psj.2026.106755

**Published:** 2026-03-10

**Authors:** Yuying Zhang, Suxin Shi, Gaoxiang Yuan, Xiaoyi Li, Zhouyang Gao, Yongfei Hu, Yuming Guo, Dan Liu

**Affiliations:** State Key Laboratory of Animal Nutrition and Feeding, College of Animal Science and Technology, China Agricultural University, Beijing 100193, China

**Keywords:** Broiler, Tryptophan, Requirement, Immune function

## Abstract

The experiment was conducted to clarify the dynamic requirement of tryptophan (Trp) in broilers under low-protein diets and construct a prediction model based on the Trp requirement. A total of 1080 male and 1080 female Arbor Acres broilers at 0 d old were randomly assigned to 6 treatments and allotted to 3 stages: 0 to 14 d starter, 15 to 28 d grower, and 29 to 42 d finisher. The birds in the control group (group NC) were fed with conventional protein diets with nutrient levels formulated to breeder recommendations. The experimental groups (groups Ⅰ to Ⅴ) were fed low-protein diets with different Trp levels which were 80% (group Ⅰ), 90% (group Ⅱ), 100% (group Ⅲ), 110% (group Ⅳ) and 120% (group Ⅴ) of NC group, respectively. The results showed that the optimal response dose of Dig.Trp/Dig.Lys was 0.172 to 0.175 for female broilers, 0.163 to 0.170 for male broilers in starter stage; 0.167 to 0.175 for female broilers, 0.168 to 0.176 for male broilers in grower stage; 0.175 to 0.177 for female broilers in finisher stage. The dynamic model of Dig.Trp/Dig.Lys daily requirement on average metabolic BW and ADG was *y* = 0.835 × BW^0.75^-1.745 × ADG-24.181 for females and *y* = -0.054 × BW^0.75^ + 0.434 × ADG-0.107 for males in starter phase; *y* = -0.208 × BW^0.75^ + 0.759 × ADG + 12.686 for females and *y* = -0.028 × BW^0.75^ + 0.523 × ADG-11.082 for males in grower phase; *y* = 0.393 × BW^0.75^-0.286 × ADG-91.961 for females and *y* = 0.146 × BW^0.75^ + 0.323 × ADG-55.300 for males in finisher phase. The optimal response Trp level in low-protein diets for growth performance can also positively regulate the development of immune organs, serum ALT, AST, immunoglobulins and hormone levels. In conclusion, optimal dietary Trp supplementation in broilers fed low-protein diets resulted in increased growth performance along with improved immune system and stress status. This research provides precise nutritional strategies to enhance poultry performance and efficiency.

## Introduction

In broiler production, the use of low-protein diets can reduce feed cost and nitrogen emission (Chrystal et al., 2020). A meta-analysis by Alfonso Avila et al. (2019) reported that each 1% CP reduction would reduce N excretion by 9%. However, with the reduction of dietary CP levels, a progression of amino acids becoming limiting for growth, making it necessary to add amino acid to meet the levels for optimal growth. Previous studies has demonstrated that reducing CP level for 1.5 to 3 percentage point would not negatively impact growth performance if supplemented with adequate types and amounts of synthetic amino acids (**AAs**) (Abou-Elkhair et al., 2020; Chrystal et al., 2020; Aderibigbe et al., 2024). On the contrary, some studies have shown that reducing dietary CP level by more than 2% may negatively affect the growth performance of broilers (Namroud et al., 2008; Kriseldi et al., 2018).

Tryptophan (**Trp**), one of the essential amino acids for poultry, has been previously reported to significantly impact feed intake, growth, behavior, immunity, and stress (Yue et al., 2017; Mund et al., 2020). Tryptophan is an important precursor for a series of bioactive substances, such as 5-hydroxytryptamine (**5-HT**), melatonin, kynurenine and nicotinamide adenine dinucleotide, etc (Linh et al., 2021). Therefore, the metabolism and nutritional functions of tryptophan are complex in the body. While the first and second limiting amino acids, lysine and methionine, have been widely added through commercial preparations, the restrictive effect of Trp is becoming increasingly prominent. At present, a considerable number of studies have reported the appropriate requirements of Trp for livestock and poultry of different ages and breeds, and it is believed that both the deficiency and excess of Trp would cause adverse effects (Rosebrough, 1996; Birkl et al., 2017). With the breeding of new broiler breeds, the improvement of feeding levels and the enhancement of feeding environments, the requirement of Trp in broiler diets is bound to change. At present, the required amounts of Trp proposed by different scholars vary greatly (Borges et al., 2016; Lee et al., 2020; Chen Jing, 2023), which means that the precise nutritional strategies are urgently needed.

Therefore, this experiment was conducted to clarify the dynamic requirement of Trp in broilers under low-protein diets and construct a prediction model based on the Trp requirement by supplementing different doses of Trp in low-protein diets.

## Materials and methods

### Animal ethics statement

The experiment procedures were in accordance with the guidelines of the Guide for the Care and Use of Agricultural Animals in Research and Teaching. The animal use and care protocol were approved by the Animal Care and Use Committee of China Agricultural University (Permit Number: AW40215202-1-03).

### Animals, diets, and experimental design

The study was conducted at the Zhuozhou experimental station of China Agricultural University. A total of 1080 male Arbor Acres broiler chicks were allotted to 3 stages: 0 to 14 d starter, 15 to 28 d grower, and 29 to 42 d finisher. Each stage utilized separate birds and birds were fed common corn-soybean meal diets prior to each experimental period that were formulated to breeder recommendations. At 0, 15 and 29 d, birds with similar body weight were randomly allocated to 6 treatments with 6 replicate pens of 10 broilers each. The birds in the control group (group NC) were fed with conventional protein diets (crude protein level was 23% in the starter phase, 21% in the grower phase, and 20% in the finisher phase). The experimental groups (groups Ⅰ to Ⅴ) were fed five low-protein diets (crude protein level was 21.5% in the starter phase, 19% in the grower phase, and 18% in the finisher phase) with different Trp levels which were 80% (group Ⅰ), 90% (group Ⅱ), 100% (group Ⅲ), 110% (group Ⅳ) and 120% (group Ⅴ) of group NC, respectively. The experimental design for female broilers was consistent with that for male broilers. The levels of Trp were combined with previous studies (Borges et al., 2016; Opoola et al., 2017; Lee et al., 2020). The composition and nutrient levels of the experimental diets were presented at Table 1. The Dig.Trp levels in experimental diets were presented at Table 2. Broilers were allowed *ad libitum* access to feed and water. The temperature and humidity were controlled automatically.

**Table 1:** Composition and nutrient levels of the experimental basal diets.

	Starter (0 to 14 d)		Grower (15 to 28 d)		Finisher (29 to 42 d)	
	NC	Ⅲ	NC	Ⅲ	NC	Ⅲ
Ingredients (%)						
Corn, 7.8% CP	48.182	54.805	52.385	59.258	52.952	59.560
Soybean meal, 43% CP	25.352	17.214	23.192	16.448	22.560	15.930
Corn gluten meal, 61%CP	9.023	9.741	6.630	5.267	5.973	5.103
Wheat flour	10.000	10.000	10.000	10.000	10.000	10.000
CaHPO_4_	1.247	1.328	1.081	1.157	0.911	0.983
Soybean oil	2.220	1.355	2.839	2.365	4.390	3.790
Limestone	1.690	1.706	1.545	1.557	1.441	1.454
Sodium chloride	0.230	0.159	0.209	0.146	0.240	0.177
50% Choline chloride	0.259	0.296	0.244	0.275	0.211	0.241
Trace mineral premix^a^	0.200	0.200	0.200	0.200	0.200	0.200
DL-Met	0.205	0.251	0.235	0.312	0.206	0.273
L-Lys HCl	0.493	0.718	0.455	0.655	0.364	0.557
Vitamin premix^b^	0.020	0.020	0.020	0.020	0.020	0.020
L-Phe	0.000	0.000	0.000	0.089	0.000	0.005
L-Thr	0.092	0.183	0.117	0.221	0.087	0.181
L-Arg	0.146	0.347	0.160	0.353	0.112	0.294
L-His	0.000	0.015	0.000	0.027	0.000	0.000
L-Ile	0.000	0.106	0.042	0.175	0.023	0.144
L-Trp	0.000	0.039	0.000	0.036	0.000	0.034
L-Val	0.059	0.168	0.079	0.211	0.042	0.161
Gly	0.075	0.300	0.088	0.343	0.000	0.100
Phytase	0.020	0.020	0.020	0.020	0.020	0.020
Zeolite	0.000	0.222	0.000	0.133	0.000	0.250
K_2_CO_3_	0.301	0.511	0.216	0.393	0.050	0.223
NaHCO₃	0.189	0.299	0.244	0.341	0.200	0.302
Total sum	100.000	100.000	100.000	100.000	100.000	100.000
Calculated composition						
Metabolizable energy (Mcal/kg)	3.050	3.050	3.100	3.100	3.200	3.200
Crude protein (%)	23.000	21.500	21.000	19.000	20.000	17.999
Calcium (%)	1.050	1.050	0.940	0.940	0.860	0.860
Non-Phytate Phosphorous (%)	0.350	0.350	0.310	0.310	0.280	0.280
Digestible Lys (%)	1.190	1.190	1.100	1.100	1.010	1.010
Digestible Met (%)	0.556	0.579	0.549	0.582	0.509	0.538
Digestible Cys (%)	0.302	0.278	0.277	0.243	0.270	0.240
Digestible Met+Cys (%)	0.857	0.857	0.825	0.825	0.778	0.778
Digestible Phe+Tyr (%)	1.538	1.416	1.378	1.265	1.329	1.162
Digestible Phe (%)	1.042	0.928	0.933	0.864	0.900	0.766
Digestible Tyr (%)	0.495	0.488	0.445	0.401	0.429	0.396
Digestible Thr (%)	0.750	0.750	0.715	0.715	0.667	0.667
Digestible Arg (%)	1.226	1.226	1.155	1.155	1.081	1.081
Digestible His (%)	0.489	0.440	0.450	0.407	0.438	0.374
Digestible Ile (%)	0.807	0.798	0.770	0.770	0.727	0.727
Digestible Leu (%)	2.085	1.972	1.838	1.576	1.763	1.548
Digestible Trp (%)	0.191	0.191	0.176	0.176	0.172	0.172
Digestible Val (%)	0.940	0.940	0.880	0.880	0.819	0.819
Digestible Gly+Ser (%)	1.750	1.750	1.617	1.617	1.486	1.354
Digestible Gly (%)	0.756	0.875	0.716	0.862	0.613	0.612
Digestible Ser (%)	0.993	0.874	0.901	0.755	0.873	0.741

a The trace mineral premix provided the following per kg of diets: Cu, 16 mg; Zn, 110 mg; Fe, 80 mg; Mn, 120 mg; Se, 0.30 mg; I, 1.50 mg.

b The vitamin premix provided the following per kg of diets: vitamin A, 15,000 IU, vitamin D3, 3,600 IU; vitamin E, 30 IU; vitamin K3, 3.00 mg; vitamin B2, 9.60 mg; vitamin B12, 0.03 mg; biotin, 0.15 mg; folic acid, 1.50 mg; pantothenic acid, 13.80 mg; nicotinic acid, 45 mg.

**Table 2:** Dig.Trp levels in experimental diets.

Items	Groups				
	Ⅰ	Ⅱ	Ⅲ	Ⅳ	Ⅴ
	Starter (0 to 14 d)				
Dig.Lys (%)	1.190	1.190	1.190	1.190	1.190
Dig.Trp (%)	0.152	0.171	0.191	0.209	0.228
Dig.Trp/Dig.Lys	0.128	0.143	0.160	0.176	0.192
	Grower (15 to 28 d)				
Dig.Lys (%)	1.100	1.100	1.100	1.100	1.100
Dig.Trp (%)	0.141	0.158	0.176	0.194	0.211
Dig.Trp/Dig.Lys	0.128	0.144	0.160	0.177	0.192
	Finisher (29 to 42 d)				
Dig.Lys (%)	1.010	1.010	1.010	1.010	1.010
Dig.Trp (%)	0.138	0.155	0.172	0.189	0.206
Dig.Trp/Dig.Lys	0.137	0.154	0.170	0.187	0.204

### Sample collection

At 14 and 28 d of the experiment, one bird per replicate with body weight close to the average of each pen was selected. At 42 d of the experiment, two birds per pen with body weight close to the average of each pen were selected. One was for sample collecting, whereas the other was for analyzing carcass traits. The selected broilers for sample collecting were stunned electrically and killed. Blood samples were collected and centrifuged at 3000 rpm for 10 min at 4 °C to obtain serum. The liver, thymus, bursa of Fabricius, and spleen were isolated. The jejunum was isolated and the middle segment of 1 to 2 cm in length was collected to analyze intestinal morphology.

### Growth performance and carcass traits

Body weight of birds per pen was measured at 0, 14, 28 and 42 d of the experiment, and feed intake per pen was measured from 0 to 14, 15 to 28, 29 to 42 d to calculate average daily feed intake (**ADFI**), average daily body weight gain (**ADG**) and feed conversion ratio (**FCR**). Birds that died during the experiment were weighed, and the data were included in the calculation of FCR.

At 42 d of the experiment, the birds selected for carcass traits were stunned electrically and killed, and then slaughtered and processed. The body weight of each bird was measured and the breast muscle and leg muscle were isolated and weighed. Dressed percentage, evisceration percentage, percentage of half-eviscerated yield, breast muscle percentage, and leg muscle percentage were calculated based on the obtained data.

### Immune organ index

At 14, 28 and 42 d, the liver, thymus, bursa of Fabricius, and spleen isolated were weighed to calculate the immune organ index using the following formula: relative weight of immune organ (g/kg) = immune organ weight (g)/body weight (kg).

### Serum parameters

In order to further verify our suggested Trp requirement level, we selected male broilers in grower stage as a representative for detecting serum biochemical levels, hormone levels and jejunal morphology. The serum contents of uric acid (**UA**), blood urea nitrogen (**BUN**), aspartate aminotransferase (**AST**), alanine aminotransferase (**ALT**), total cholesterol (**TC**), total triglyceride (**TG**), total protein (**TP**), albumin (**ALB**), immunoglobulin G (**IgG**), immunoglobulin A (**IgA**), and immunoglobulin M (**IgM**) were determined using an automatic biochemical analyzer (Kehua ZY KHB-1280). The levels of cholecystokinin (**CCK**), Ghrelin, adrenocorticotropic hormone (**ACTH**), and corticosterone (**CORT**) were determined by enzyme-linked immunosorbent assay kits (ML061003, ML060906, ML061155, and ML059881, MLBIO, respectively).

### Jejunum morphology

The jejunal samples collected were fixed in 4% Paraformaldehyde and embedded in paraffin. One section was cut per sample, stained with hematoxylin-eosin, and observed and analyzed by the Leika DM750 microscope (Wetzlar, Germany) connected to the computer. Ten complete intestinal villi were randomly selected from each section to measure villus height, crypt depth, and calculate the ratio of villus height to crypt depth (**V/C**).

### Statistical analysis

All data were analyzed by one-way analysis of variance (**ANOVA**) using SPSS version 26.0. Differences among treatments were examined using Duncan's multiple range tests, which were considered significant at *P* < 0.05.

Data were analyzed for linear and quadratic response using regression. The linear regression model was expressed as, $y_{i}=ax_{i}+b$, where $y_{i}$ is the dependent variable, $x_{i}$ is the dietary Dig.Trp/Dig.Lys concentration, and b is the intercept, a is the linear coefficient. The quadratic polynomial (**QP**) regression model was expressed as, $y_{i}=ax_{i}^{2}+bx_{i}+c$, where $y_{i}$ is the dependent variable, $x_{i}$ is the dietary Dig.Trp/Dig.Lys concentration, and c is the intercept, a and b are the quadratic and linear coefficients, respectively. Dependent variables in the present study included FBW, ADG, ADFI, FCR and dressed percentage. The QP regression model was fitted to estimate the optimal level of Dig.Trp/Dig.Lys to maximize broiler growth performance and carcass trait data. The maximum responses were obtained by −b÷(2×a).

Additionally, in order to give out a dynamic feeding strategy based on body weight, a multiple linear regression model was conducted with the average metabolic body weight (BW^0.75^) and ADG as the independent variables, and the Dig.Trp/Dig.Lys daily intake as the dependent variable. The Dig.Trp/Dig.Lys daily intakes were obtained by dietary Dig.Trp/Dig.Lys level multiply ADFI.

## Results

### Optimal tryptophan levels on growth performance and carcass traits

**Fig. 1:**
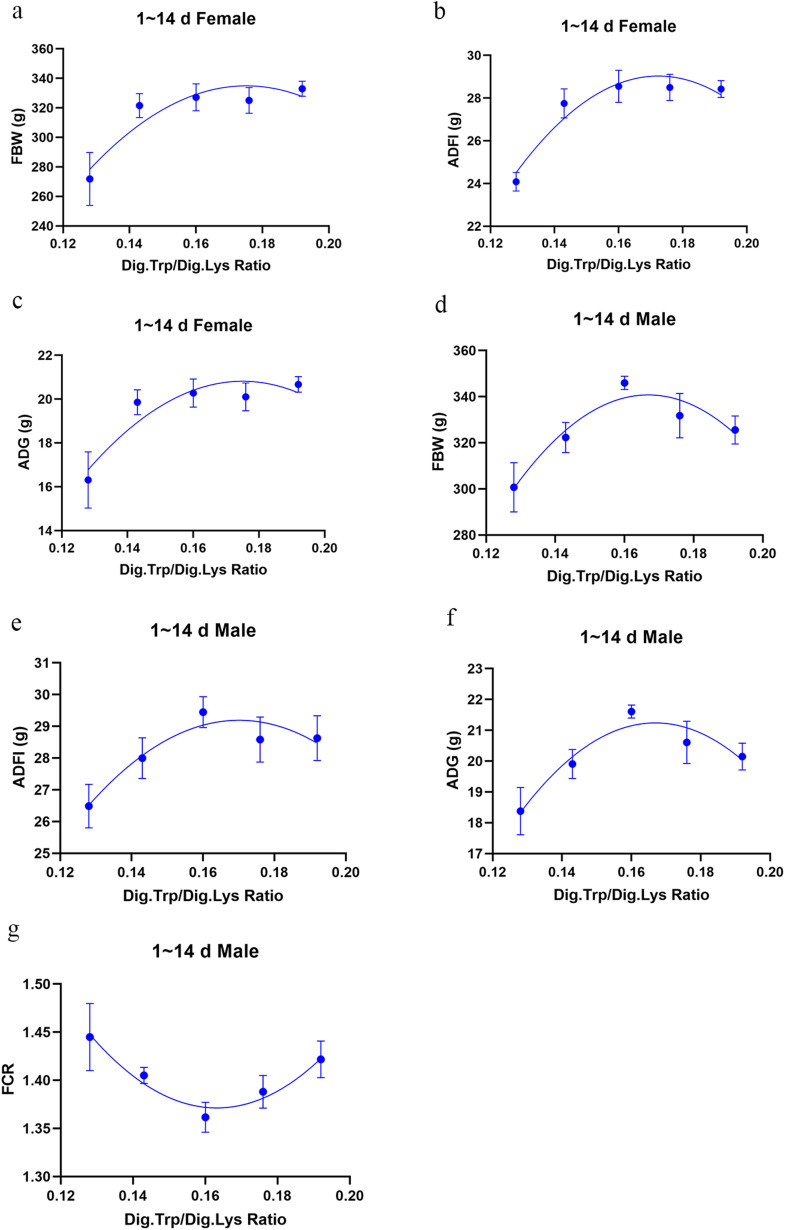
Experimental responses of growth performance to dietary Dig.Trp/Dig.Lys concentrations in broilers from 1 to 14 days of age.

**Fig. 2:**
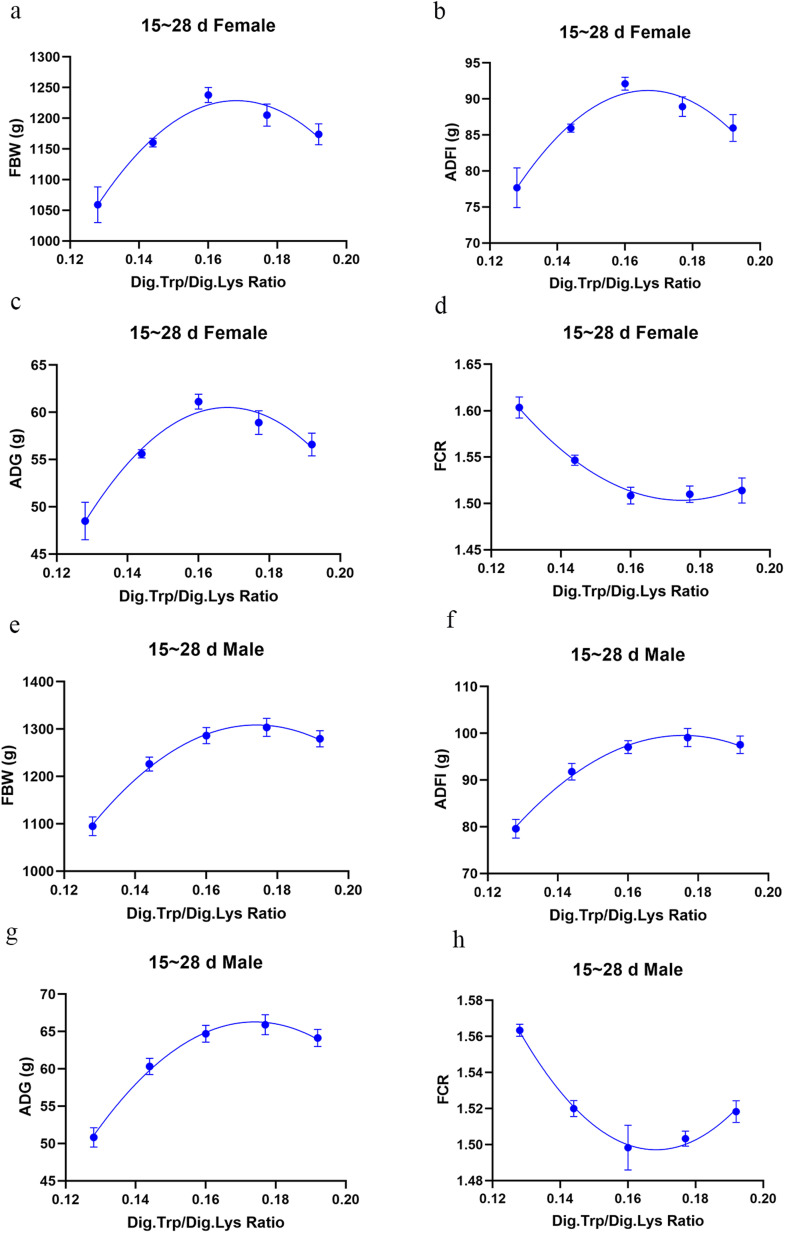
Experimental responses of growth performance to dietary Dig.Trp/Dig.Lys concentrations in broilers from 15 to 28 days of age.

**Fig. 3:**
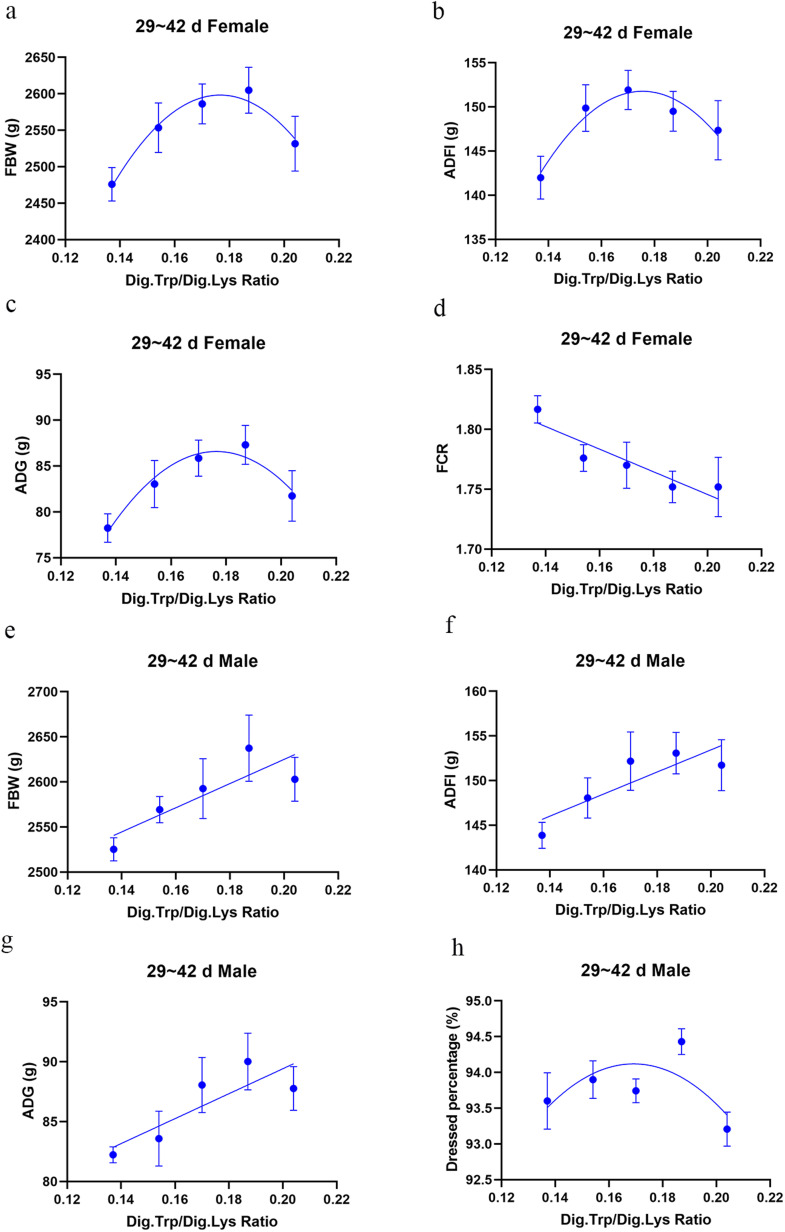
Experimental responses of growth performance and carcass trait to dietary Dig.Trp/Dig.Lys concentrations in broilers from 29 to 42 days of age.

Influence of dietary treatments on the growth performance of broilers are shown in Table 3. The regression curve and Dig.Trp/Dig.Lys requirements for growth performance and carcass traits data are shown in Table 5 and Fig. 1, Fig. 2, Fig. 3. In the starter phase, there was a significant quadratic change in all indicators of growth performance except for the FCR of female broilers with the increase of Trp levels in low protein diets (*P* < 0.05). As for female broilers, the relevant quadratic regressions predicted that FBW and ADG would deteriorate from a maximum once the ratio of Dig.Trp/Dig.Lys exceeds 0.175, and the maximum ADFI corresponded to a 0.172 ratio. As for male broilers, the relevant quadratic regressions predicted that FBW and ADG would achieve a maximum once the ratio of Dig.Trp/Dig.Lys reaches 0.167, and the maximum ADFI corresponded to a 0.170 ratio. In the grower phase, we noticed that compared with NC group, the FCR of both female and male broilers was significantly increased in low-protein diet groups (*P* < 0.05). There was a significant quadratic change in all indicators of growth performance as the Trp levels increased in low protein diets (*P* < 0.05). As for female broilers, the relevant quadratic regressions predicted that the maximum FBW and ADG corresponded to a 0.168 Dig.Trp/Dig.Lys ratio, and the maximum ADFI corresponded to a 0.167 ratio. As for male broilers, the maximum FBW, ADG and ADFI separately corresponded to 0.174, 0.175 and 0.176 Dig.Trp/Dig.Lys ratio. In the finisher phase, we noticed that decreasing CP level by 2% didn’t affect the growth performance of both female and male broilers. Increasing dietary Trp concentrations in low-protein diets linearly increased the FBW, ADFI and ADG of male broilers (*P* < 0.05) and decreased the FCR of female broilers (*P* < 0.05). As the Trp levels increased in low protein diets, there was a highly significant quadratic change in FBW, ADFI and ADG of female broilers (*P* < 0.05), predicting the optimal response dose as 0.177, 0.175 and 0.176 Dig.Trp/Dig.Lys ratio.

**Table 3:** Growth performance of Arbor Acre broilers fed diets varying in digestible tryptophan supplementation from 0 to 14, 15-28 and 29-42 days of age.

	Female					Male			
Groups	FBW (g)	ADFI (g)	ADG (g)	FCR (g:g)		FBW (g)	ADFI (g)	ADG (g)	FCR (g:g)
	0 to 14 d of age								
NC	351.12^a^	28.73^a^	21.97^a^	1.31^b^		348.83^a^	29.67^a^	21.83^a^	1.36
Ⅰ	271.84^b^	24.09^b^	16.31^b^	1.45^a^		300.72^c^	26.49^b^	18.38^d^	1.45
Ⅱ	321.53^a^	27.75^a^	19.86^a^	1.40^ab^		322.32^b^	28.00^ab^	19.91^cd^	1.41
Ⅲ	327.16^a^	28.55^a^	20.27^a^	1.41^ab^		345.93^a^	29.45^a^	21.61^ab^	1.36
Ⅳ	325.08^a^	28.50^a^	20.11^a^	1.42^a^		331.78^ab^	28.58^a^	20.61^abc^	1.39
Ⅴ	332.98^a^	28.42^a^	20.67^a^	1.38^ab^		325.55^ab^	28.63^a^	20.15^bc^	1.42
SEM	5.69	0.35	0.41	0.01		3.95	0.30	0.28	0.01
*P-*value^1^	<0.001	<0.001	<0.001	0.049		0.007	0.019	0.007	0.074
Linear^2^	0.001	<0.001	0.001	0.282		0.020	0.025	0.019	0.349
Quadratic^2^	0.031	0.001	0.030	0.971		0.003	0.040	0.003	0.011
	15 to 28 d of age								
NC	1260.72^a^	89.46^ab^	62.90^a^	1.42^d^		1334.67^a^	96.18^ab^	67.93^a^	1.42^c^
Ⅰ	1059.17^d^	77.69^c^	48.50^d^	1.60^a^		1094.92^c^	79.59^c^	50.83^d^	1.57^a^
Ⅱ	1160.28^c^	85.96^b^	55.61^c^	1.55^b^		1226.12^b^	91.78^b^	60.33^c^	1.52^b^
Ⅲ	1237.76^ab^	92.11^a^	61.13^ab^	1.51^c^		1286.33^a^	97.07^ab^	63.66^bc^	1.50^b^
Ⅳ	1205.03^bc^	88.92^ab^	58.91^bc^	1.51^c^		1303.49^a^	99.08^a^	65.91^ab^	1.50^b^
Ⅴ	1173.93^c^	85.96^b^	56.59^c^	1.51^c^		1279.65^a^	97.56^a^	64.14^b^	1.52^b^
SEM	12.97	1.00	0.91	0.01		14.93	1.32	1.07	0.01
*P-*value^1^	<0.001	<0.001	<0.001	<0.001		<0.001	<0.001	<0.001	<0.001
Linear^2^	<0.001	0.001	<0.001	<0.001		<0.001	<0.001	<0.001	<0.001
Quadratic^2^	<0.001	<0.001	<0.001	<0.001		<0.001	<0.001	<0.001	<0.001
	29 to 42 d of age								
NC	2591.98	148.38	85.72	1.73^b^		2588.55	147.41	86.98	1.69
Ⅰ	2475.92	141.99	78.24	1.82^a^		2525.28	143.88	82.24	1.75
Ⅱ	2553.52	149.88	83.04	1.78^ab^		2569.33	148.06	83.58	1.78
Ⅲ	2586.10	151.92	85.86	1.77^ab^		2592.60	152.18	88.06	1.71
Ⅳ	2604.80	149.52	87.31	1.75^b^		2637.47	153.07	90.02	1.71
Ⅴ	2531.67	147.35	81.74	1.75^b^		2602.93	151.73	87.77	1.73
SEM	14.08	1.14	1.01	0.01		11.43	1.11	0.85	0.01
*P-*value^1^	0.069	0.186	0.088	0.037		0.093	0.122	0.059	0.194
Linear^2^	0.110	0.221	0.123	0.009		0.014	0.015	0.010	0.104
Quadratic^2^	0.012	0.019	0.014	0.276		0.186	0.140	0.205	0.544

a-d Means within a column without a common superscript differ significantly (*P* < 0.05).

1 Overall *P*-values obtained from ANOVA.

2 *P*-values obtained using contrast trend analysis.

The effects of dietary treatments on carcass traits at 42 d are reported in Table 4. Dietary protein level and Trp concentrations did not affect the carcass traits of female broilers. For male broilers, with the increase of Trp levels in low protein diets, the dressed percentage showed a quadratic change of first improve and then reduce (*P* = 0.04). Furthermore, when Dig.Trp/Dig.Lys ratio reached 0.187 (group Ⅳ), the dressed percentage was significantly higher than broilers fed the conventional protein diet (*P* < 0.05).

**Table 4:** Carcass traits of Arbor Acre broilers fed diets varying in digestible tryptophan supplementation from 29 to 42 days of age.

	Items^3^				
Groups	DP (%)	PHY (%)	EP (%)	BMP (%)	LMP (%)
	Female				
NC	94.37	88.62	77.38	28.19	18.74
Ⅰ	93.82	88.98	77.76	28.82	19.61
Ⅱ	93.89	89.42	77.88	26.54	19.96
Ⅲ	93.77	89.08	77.64	28.86	19.00
Ⅳ	94.40	90.02	78.11	28.67	18.64
Ⅴ	94.05	89.31	78.61	27.70	19.05
SEM	0.17	0.18	0.20	0.26	0.21
*P-*value^1^	0.654	0.317	0.587	0.058	0.444
Linear^2^	0.355	0.335	0.248	0.952	0.178
Quadratic^2^	0.941	0.520	0.456	0.961	0.731
	Male				
NC	93.07^b^	88.20	76.32	27.26	19.88
Ⅰ	93.60^ab^	88.36	76.01	26.68	20.06
Ⅱ	93.90^ab^	89.18	76.61	25.62	19.89
Ⅲ	93.74^ab^	89.03	77.24	26.22	20.82
Ⅳ	94.43^a^	89.64	77.17	25.47	19.52
Ⅴ	93.21^b^	88.88	76.44	25.28	20.81
SEM	0.14	0.18	0.23	0.37	0.09
*P-*value^1^	0.032	0.212	0.658	0.618	0.347
Linear^2^	0.757	0.308	0.464	0.349	0.439
Quadratic^2^	0.040	0.182	0.159	0.915	0.700

a,b Means within a column without a common superscript differ significantly (*P* < 0.05).

1 Overall *P*-values obtained from ANOVA.

2 *P*-values obtained using contrast trend analysis.

3 DP, dressed percentage; PHY, percentage of half-eviscerated yield; EP, evisceration percentage; BMP, breast muscle percentage; LMP, leg muscle percentage.

**Table 5:** Dig.Trp/Dig.Lys requirement of Arbor Acre broilers fed low protein diets from 0 to 14, 15-28 and 29-42 days of age.

Age	Parameter	Sex	Equation	R^2^	SSE^1^	*P*-value	Requirement
0-14 d			Quadratic polynomial (QP)				
	FBW	Female	−25459x^2^ + 8918.3x-446.1	0.401	26.630	0.001	0.175
		Male	−26845x^2^ + 8960.4x-406.9	0.410	18.067	0.001	0.167
	ADFI	Female	−2295.7x^2^ + 791.53x-39.2	0.552	1.480	<0.001	0.172
		Male	−1511.6x^2^ + 514.25x-14.55	0.299	1.538	0.010	0.170
	ADG	Female	−1829x^2^ + 640.18x-35.2	0.401	1.902	0.001	0.175
		Male	−1920.6x^2^ + 641.13x-32.27	0.411	1.290	0.001	0.167
	FCR	Male	61.838x^2^-20.181x + 3.018	0.261	0.050	0.020	0.163
15-28 d			Quadratic polynomial (QP)				
	FBW	Female	−105575x^2^ + 35503x-1756.2	0.664	44.540	<0.001	0.168
		Male	−98809x^2^ + 34413x-1687.8	0.785	41.920	<0.001	0.174
	ADFI	Female	−9018.8x^2^ + 3007.4x-159.54	0.617	3.988	<0.001	0.167
		Male	−8582.2x^2^ + 3016.6x-165.53	0.755	4.286	<0.001	0.176
	ADG	Female	−7494.4x^2^ + 2520.4x-151.4	0.681	3.043	<0.001	0.168
		Male	−6663x^2^ + 2333.5x-138.38	0.817	2.716	<0.001	0.175
	FCR	Female	45.574x^2^-15.922x + 2.894	0.735	0.023	<0.001	0.175
		Male	41.20x^2^-13.854x + 2.662	0.717	0.015	<0.001	0.168
29-42 d			Quadratic polynomial (QP)				
	FBW	Female	−80293x^2^ + 28355x + 94.98	0.285	73.859	0.011	0.177
	ADFI	Female	−6237.4x^2^ + 2189.1x-40.307	0.236	6.214	0.026	0.175
	ADG	Female	−5628.03x^2^ + 1986.51x-88.708	0.272	5.325	0.014	0.176
	Dressed percentage	Male	−591.55x^2^ + 200.16x + 77.187	0.153	0.693	0.115	0.169
			Linear polynominal (LP)				
	FBW	Male	1340.3x + 2356.9	0.208	64.506	0.013	-
	ADFI	Male	123.48x + 128.74	0.198	6.082	0.014	-
	ADG	Male	104.15x + 68.586	0.212	4.909	0.010	-
	FCR	Female	−0.945x + 1.935	0.272	0.038	0.005	-

1 SSE, sum of squared errors.

### Tryptophan dynamic requirement model

In order to give out guidance for precision feeding, the regression model of Dig.Trp/Dig.Lys daily intake on average metabolic BW and ADG was presented in Table 6. The dynamic model of Dig.Trp/Dig.Lys daily requirement on average metabolic BW and ADG was *y* = 0.835 × BW^0.75^-1.745 × ADG-24.181 for females and *y* = −0.054 × BW^0.75^ + 0.434 × ADG-0.107 for males in starter phase; *y* = −0.208 × BW^0.75^ + 0.759 × ADG + 12.686 for females and *y* = −0.028 × BW^0.75^ + 0.523 × ADG-11.082 for males in grower phase; *y* = 0.393 × BW^0.75^-0.286 × ADG-91.961 for females and *y* = 0.146 × BW^0.75^ + 0.323 × ADG-55.300 for males in finisher phase.

**Table 6:** Regression equation of Dig.Trp/Dig.Lys with body weight and body weight gain.

Age	Regression equation	R^2^
0-14 d	Female *y* = 0.835 × BW^0.75^ − 1.745 × ADG-24.181	0.511
	Male *y* = −0.054 × BW^0.75^ + 0.434 × ADG-0.107	0.367
15-28 d	Female *y* = −0.208 × BW^0.75^ + 0.759 × ADG + 12.686	0.634
	Male *y* = −0.028 × BW^0.75^ + 0.523 × ADG-11.082	0.802
29-42 d	Female *y* = 0.393 × BW^0.75^-0.286 × ADG-91.961	0.220
	Male *y* = 0.146 × BW^0.75^ + 0.323 × ADG-55.300	0.402

### Effects of tryptophan supplementation on immunity organ index

Influence of different dietary treatments on the immune organ index of broilers are presented in Table 7. For female broilers, decreasing the protein level increased the thymus index in the grower stage, for the thymus index in Ⅰ and Ⅴ group were significantly higher than in NC group (*P* < 0.05). For male broilers, decreasing the protein level for 1.5% to 2% points increased the liver index, especially in the starter and finisher stage. In the starter stage, the liver index of broilers in Ⅳ and Ⅴ group were significantly higher than in NC group (*P* < 0.05). In the finisher stage, the liver index of broilers in Ⅱ and Ⅴ group were significantly higher than in NC group (*P* < 0.05).

**Table 7:** Immune organ index of Arbor Acre broilers fed diets varying in digestible tryptophan supplementation from 0 to 14, 15 to 28, and 29 to 42 days of age.

	Female					Male			
Groups	Liver index	Spleen index	Bursa of Fabricius index	Thymus index		Liver index	Spleen index	Bursa of Fabricius index	Thymus index
	0 to 14 d of age								
NC	43.95	0.80	1.71	3.68		40.06^c^	0.93	2.40	4.68
Ⅰ	44.97	0.94	2.05	4.68		42.74abc	0.91	2.20	5.75
Ⅱ	48.25	0.70	1.85	4.27		41.71^bc^	0.82	2.21	5.63
Ⅲ	49.86	0.68	1.78	4.24		45.78^abc^	0.72	2.37	4.41
Ⅳ	46.49	0.66	1.83	4.91		48.75^a^	0.68	2.10	4.50
Ⅴ	46.44	0.67	2.01	4.91		46.79^ab^	0.81	2.25	4.47
SEM	0.95	0.03	0.05	0.14		0.92	0.04	0.09	0.18
*P-*value^1^	0.533	0.126	0.341	0.082		0.022	0.348	0.952	0.075
Linear^2^	0.865	0.029	0.773	0.296		0.033	0.249	0.998	0.015
Quadratic^2^	0.170	0.752	0.047	0.228		0.711	0.152	0.862	0.365
	15 to 28 d of age								
NC	30.33	1.02	1.76	4.99^c^		28.83	0.92^b^	1.97^a^	5.76
Ⅰ	33.22	1.09	1.97	7.44^a^		31.75	1.22^ab^	2.23^a^	5.31
Ⅱ	32.45	1.03	1.80	6.14^bc^		29.59	1.23^ab^	1.92^a^	6.55
Ⅲ	33.92	0.89	1.47	6.24abc		33.03	0.96^b^	2.06^a^	4.69
Ⅳ	30.62	0.98	2.02	5.99^bc^		28.84	0.88^b^	2.15^a^	4.52
Ⅴ	33.00	1.01	1.80	6.70^ab^		33.77	1.44^a^	1.25^b^	5.00
SEM	0.65	0.04	0.07	0.20		0.63	0.06	0.10	0.25
*P-*value^1^	0.541	0.812	0.256	0.001		0.085	0.014	0.025	0.175
Linear^2^	0.629	0.495	0.785	0.217		0.478	0.871	0.015	0.185
Quadratic^2^	0.781	0.284	0.187	0.022		0.235	0.027	0.130	0.945
	29 to 42 d of age								
NC	29.34	1.07	1.55	4.52		23.35^b^	1.16^a^	1.65	4.24
Ⅰ	28.14	1.10	1.66	4.80		25.77^ab^	1.06^ab^	1.33	4.07
Ⅱ	28.07	1.01	2.12	4.75		27.92^a^	1.06^ab^	1.95	5.02
Ⅲ	28.42	0.93	1.70	4.67		26.38^ab^	0.88^ab^	1.59	4.38
Ⅳ	28.63	0.99	1.60	4.70		26.60^ab^	0.80^b^	1.43	4.16
Ⅴ	28.83	0.91	1.88	4.30		27.81^a^	0.89^ab^	1.80	5.10
SEM	0.45	0.04	0.08	0.17		0.47	0.04	0.08	0.13
*P-*value^1^	0.976	0.736	0.318	0.973		0.038	0.018	0.184	0.056
Linear^2^	0.607	0.226	0.900	0.479		0.412	0.013	0.494	0.235
Quadratic^2^	0.929	0.630	0.945	0.742		0.978	0.286	0.657	0.724

a-c Means within a column without a common superscript differ significantly (*P* < 0.05).

1 Overall *P*-values obtained from ANOVA.

2 *P*-values obtained using contrast trend analysis.

For female broilers, as Trp level increased in low-protein diets, the spleen index decreased linearly (*P* = 0.029), the bursa of Fabricius index showed a quadratic change (*P* = 0.047) of first decline and then increase in the starter stage. In the grower stage, the thymus index showed a quadratic change (*P* = 0.022) of first decrease and then increase. For male broilers, as Trp level increased in low-protein diets, the liver index increased linearly (*P* = 0.033), the thymus index decreased linearly (*P* = 0.015) in the starter stage. The spleen index showed a quadratic change (*P* = 0.027) of first decrease and then increase in the grower stage, decreased linearly (*P* = 0.013) in the finisher stage. The bursa of Fabricius index decreased linearly (*P* = 0.015) in the grower stage.

### Effects of tryptophan supplementation on blood biochemical and hormone levels, and jejunum morphology

In order to find out whether the optimal response Trp level for growth performance can also positively regulate the serum parameters and jejunal morphology, we chose male broilers in grower stage as a representative. The effects of different dietary treatments on the serum biochemical levels of male broilers in grower stage are shown in Table 8. As Trp level increased in low-protein diets, the levels of serum ALT, AST and TG decreased linearly (*P* < 0.05), the level of UA showed a quadratic change of first decreasing and then increasing (*P* = 0.044), and the level of GLU increased linearly (*P* = 0.001). Serum immunoglobulin content was also significantly affected by different dietary treatments. The levels of serum IgG, IgA and IgM linearly decreased with the increase of Trp level in low-protein diets (*P* < 0.05).

**Table 8:** Serum biochemical levels of Arbor Acre broilers fed diets varying in digestible tryptophan supplementation from 15 to 28 days of age.

	Items^3^											
Groups	TP (g/L)	ALB (g/L)	ALT (U/L)	AST (U/L)	TC (mmol/L)	TG (mmol/L)	BUN (mmol/L)	UA (umol/L)	GLU (mmol/L)	IgG (g/L)	IgA (g/L)	IgM (g/L)
NC	25.92	18.14	53.04^a^	209.70^a^	4.02	1.01^b^	1.76	163.73	9.59^bc^	4.25^a^	2.28^a^	1.67
Ⅰ	26.82	19.18	49.41^ab^	217.30^a^	4.18	1.11^b^	1.75	169.24	8.88^c^	4.23^a^	2.31^a^	1.66
Ⅱ	25.89	18.65	49.02^ab^	212.49^a^	4.13	1.52^a^	1.83	165.14	9.96^abc^	4.23^a^	2.29^a^	1.66
Ⅲ	25.71	18.82	48.90^ab^	194.15^ab^	4.3	1.06^b^	1.75	154.63	8.92^c^	4.15^ab^	2.22^ab^	1.63
Ⅳ	26.62	18.08	45.60^bc^	197.22^ab^	4.46	1.01^b^	1.68	163.07	10.74^ab^	4.22^a^	2.26^a^	1.65
Ⅴ	27.79	18.42	43.53^c^	172.13^b^	4.29	0.94^b^	1.83	178.64	11.17^a^	4.10^b^	2.16^b^	1.59
SEM	0.40	0.30	0.80	4.33	0.07	0.06	0.07	2.97	0.22	0.02	0.01	0.01
*P-*value^1^	0.703	0.916	0.006	0.020	0.496	0.028	0.993	0.324	0.003	0.023	0.021	0.106
Linear^2^	0.424	0.425	0.004	0.001	0.221	0.048	0.981	0.479	0.001	0.008	0.002	0.027
Quadratic^2^	0.187	0.788	0.253	0.580	0.600	0.262	0.827	0.044	0.359	0.423	0.676	0.502

a-c Means within a column without a common superscript differ significantly (*P* < 0.05).

1 Overall *P*-values obtained from ANOVA.

2 *P*-values obtained using contrast trend analysis.

3 TP, total protein; ALB, albumin; ALT, alanine aminotransferase; AST, aspartate aminotransferase; TC, total cholesterol; TG, total triglyceride; BUN, blood urea nitrogen; UA, uric acid; IgG, immunoglobulin G; IgA, immunoglobulin A; IgM, immunoglobulin M.

The effects of different dietary treatments on the serum hormone levels of male broilers in grower stage are shown in Table 9. With the increase of Trp levels in low-protein diets, both CCK and Ghrelin decreased linearly (*P* < 0.05). Different dietary treatments significantly changed the levels of serum ACTH and CORT (*P* < 0.05). As Trp level in low-protein diets increased, the concentration of ACTH decreased linearly (*P* = 0.001), and the level of CORT showed a quadratic curve change of first decreasing and then increasing (*P* = 0.001).

**Table 9:** Hormone levels of Arbor Acre broilers fed diets varying in digestible tryptophan supplementation from 15 to 28 days of age.

	Items^3^			
Groups	CCK (pg/mL)	Ghrelin (pg/mL)	ACTH (pg/mL)	CORT (ng/mL)
NC	41.71^ab^	133.86^ab^	11.82^ab^	26.21^ab^
Ⅰ	44.83^a^	141.83^a^	12.46^a^	24.67^bc^
Ⅱ	41.05^abc^	129.91^abc^	11.22^abc^	21.20^c^
Ⅲ	44.75^a^	142.00^a^	12.36^a^	23.57^bc^
Ⅳ	37.76^bc^	120.47^bc^	10.45^bc^	23.15^bc^
Ⅴ	36.77^c^	116.13^c^	9.78^c^	29.18^a^
SEM	0.78	2.61	0.25	0.60
*P-*value^1^	0.006	0.018	0.002	0.002
Linear^2^	0.001	0.002	0.001	0.007
Quadratic^2^	0.394	0.372	0.311	0.001

a-c Means within a column without a common superscript differ significantly (*P* < 0.05).

1 Overall *P*-values obtained from ANOVA.

2 *P*-values obtained using contrast trend analysis.

3 CCK, cholecystokinin; ACTH, adrenocorticotropic hormone; CORT, corticosterone.

Then we analyzed jejunal morphology of male broilers in grower stage (Table 10), finding that as Trp level increased in low-protein diets, villus height showed a tendency of linearly increasing (*P* = 0.099).

**Table 10:** Jejunum morphology of Arbor Acre broilers fed diets varying in digestible tryptophan supplementation from 15 to 28 days of age.

	Items^3^		
Groups	villus height (µm)	crypt depth (µm)	V/C
NC	998.07	217.53	4.82
Ⅰ	1016.47	201.77	5.11
Ⅱ	938.11	199.14	5.32
Ⅲ	1096.52	196.76	5.46
Ⅳ	1002.45	200.89	5.29
Ⅴ	1182.17	207.64	5.89
SEM	31.79	4.64	0.17
*P-*value^1^	0.281	0.917	0.068
Linear^2^	0.099	0.690	0.249
Quadratic^2^	0.338	0.540	0.761

1 Overall *P*-values obtained from ANOVA.

2 *P*-values obtained using contrast trend analysis.

3 V/C, the ratio of villus height to crypt depth.

## Discussion

It is expected that broilers offered appropriate Trp had better growth performance. In present experiment, through all three stages, as Trp level increased in low-protein diets, the growth performance of broilers showed a trend of first increasing and then decreasing. In starter and grower stage, either the deficiency or excess of Trp in low-protein diets significantly affected the growth performance of broilers. Moreover, while the Trp level reached the optimal response dose, it could only make the growth performance of broilers statistically consistent with that of the conventional protein diet group. In finisher stage, compared with the NC group, except that feeding broilers the low-protein diet with the lowest level of Trp significantly increased the FCR, there were no significant differences in other indicators of production performance.

In the present study, we found that reducing Trp levels significantly affected the feed intake of broilers, especially in the starter and grower stages. This result was consistent with Birkl et al. (2017), who pointed out that Trp deficiency led to the decrease of Trp levels in plasma, negatively affecting the synthesis of the neurotransmitter serotonin, thereby reducing the feed intake of poultry and even causing behaviors such as feather pecking. However, excessively increasing the Trp level in low-protein diets might cause negative effect on the feed intake, being in agreement with what Xie et al. (2024) reported. Ghrelin is a hormone that promotes appetite in mammals, but oppositely suppresses appetite in poultry (Kaiya et al., 2009). Similarly, CCK would suppress appetite by stimulating the hypothalamus to produce satiety signals (Te Pas et al., 2020). In our study, the concentrations of serum Ghrelin and CCK linearly declined as dietary Trp increased, indicating that Trp could promote appetite in broiler, in line with the promotion of feed intake.

Aderibigbe et al. (2024) found that reducing the dietary CP level by approximately 3% had no effect on the weight and ratio of carcass, breast muscle, and leg muscle of broilers. van Harn et al. (2019) reported that reducing dietary CP level for one or two percentage point did not have negative effect on slaughter yields of broilers. These results were similar to our experiment, in which reducing the dietary crude protein level by 2% had no significant effect on the evisceration percentage, percentage of half-eviscerated yield, breast muscle percentage, leg muscle percentage of male and female broilers. This indicated that as long as the adequate amount of animo acid were added, reducing the CP level in the diet would not have a negative impact on the slaughter performance of broilers. Moreover, in our experiment, the dressed percentage of male broilers showed a significant quadratic curve change with the increase of Trp level in the low-protein diets. Consistent with previous research (Corzo et al., 2005), this indicated that an appropriate level of Trp is conducive to the optimal slaughter performance of broilers.

According to growth performance and carcass traits, the optimal response dose of Trp was given out. In starter phase, the recommended ratio of Dig.Trp/Dig.Lys for female broilers was 0.172 to 0.175; that for male broilers was 0.163 to 0.170. In grower phase, the recommended ratio of Dig.Trp/Dig.Lys for female broilers was 0.167 to 0.175; that for male broilers was 0.168 to 0.176. In finisher phase, the recommended ratio of Dig.Trp/Dig.Lys for female broilers was 0.175 to 0.177. These recommended doses were higher than current standard-recommended Trp requirements, suggesting that Trp level should be appropriately increased when using low-protein diets. Moreover, it can be noted that female broilers require more Trp than male broilers in starter phase, while in grower phase, the requirement of male broilers exceeds that of females. This may due to a potential difference in nutrient uptake in the intestine between male and female broilers, which is consistent with the results that male and female broilers differ in their growth performance (England et al., 2024). This gender difference further emphasizes the necessity of precision feeding, which can enhance the overall production performance of the flock and reduce feed costs.

In order to further verify our suggested Trp requirement level, we chose male broilers in grower stage as a representative for detecting serum biochemical levels, hormone levels and jejunal morphology, leading to a conclusion that whether the optimal response Trp level for growth performance can also positively regulate the immune system and hormone levels.

It was suggested that reducing dietary protein level would negatively affect the immune status of broilers. By evaluating the immune organ index, a comprehensive assessment of the immune status of broilers can be conducted. Previous studies have shown that feeding low-protein diets might lead to excessive fat supply, affecting liver metabolism in broiler and subsequently causing the occurrence of fatty liver (Heo et al., 2023). In present research, we found that compared with NC group, broilers feeding low-protein diets presented higher liver index, which might be due to liver hypertrophy or hyperplasia. ALT and AST are important markers for assessing liver damage. In present experiment, increasing the Trp levels in low-protein diets linearly decreased serum ALT and AST concentrations, suggesting that adding adequate Trp would alleviate the liver damage caused by low-protein diets. In accordance with our result, El-Kholy et al. (2025) found that adding Trp to quail diets significantly decreased the activity of liver enzymes including ALT and AST. Thymus is a center of the immune system, where T cells develop and mature (Gordon and Manley, 2011). In present experiment, broilers fed low-protein diets with relatively low or high Trp levels tended to have higher thymus index, especially in starter and grower phase of female broilers. As Trp concentrations increased in low-protein diets, the thymus index of male broilers in starter phase decreased linearly, and that of female broilers in grower phase showed a quadratic change of first decreasing and then increasing. Furthermore, when the Trp levels were 90% to 110% of NC group, the thymus index wouldn’t be significantly affected by low-protein diets. This indicated that reducing dietary protein levels by 1.5% to 2% points may not significantly change the immune status if adequate amount of Trp added. The result of serum immunoglobulins cohered with this, which showed that serum immunoglobulins wouldn’t be significantly affected by low-protein diets. Furthermore, the concentrations of IgA, IgG and IgM decreased linearly with the increase of Trp levels. However, Dong et al. (2012) found that supplementing Trp in the diet of laying hens reared under hot and humid summer conditions quadratically increased serum IgM. Also, the experiment on broilers with infectious bursal disease showed that Trp could increase the level of serum IgG, thereby helping the broilers recover from disease (Emadi et al., 2011). In that, we suggested that if under stress or suffered from inflammation, the Trp has a positive effect on immune responses; if under normal physiological conditions, our suggesting Trp dosages are enough.

It has been reported that supplementing Trp can improve intestinal structure by stimulating the regeneration of intestinal villi and crypt cells in weaned piglets (Koopmans et al., 2006). In present experiment, the jejunal villi height in 28-day-old male broilers showed a linear increasing trend with the increase of Trp levels in low-protein diets. However, different dietary treatments didn’t have significant influence on crypt depth or villus-crypt ratio. This might due to the fact that in present experiment broilers were under normal physiological conditions and thus the effect of dietary Trp levels on intestinal structure could not be demonstrated.

It has been shown that Trp can regulate the secretion of corticosterone, cortisol and 5-HT under normal physiological conditions, and these hormones determine the stress state of broilers (Fouad et al., 2021). Trp can reduce the aggressive behavior of birds by increasing the level of 5-HT in the central nervous system. 5-HT can regulate the hypothalamic-pituitary-adrenal axis and therefore affect the secretion of glucocorticoids (Xue et al., 2023). Studies have shown that the over-activation of the hypothalamic-pituitary-adrenal axis can lead to an increase in corticotropin-releasing hormone and adrenocorticotropic hormone, resulting in emotional and cognitive disorders (Gordon and Manley, 2011). In this experiment, as the level of Trp in low-protein diets increased, the level of ACTH decreased linearly, and the level of CORT showed a quadratic curve change of first decreasing and then increasing, indicating that an appropriate level of Trp is conducive.

The serum UA level reflects the situation of protein metabolism and amino acid balance in broilers (Cappelaere et al., 2021). Previous studies suggested that dietary CP reduction with balanced amino acid content lowers the supply of excess amino acid, which reduces the amino acid catabolism as evidenced by the reduction of plasma UA concentration (Namroud et al., 2008; Kriseldi et al., 2018). However, in present experiment, reducing dietary CP level by 2% didn’t significantly affect serum UA concentration. Our results showed that the serum UA levels showed a quadratic curve change of first decreasing and then increasing with the increase of Trp level in low-protein diets. This indicated that an appropriate level of Trp is beneficial for amino acid metabolism in broilers and can reduce nitrogen excretion.

## Conclution

The experiment was conducted to clarify the dynamic requirement of Trp in broilers under low-protein diets and construct a prediction model based on the Trp requirement. The results showed that the optimal response dose of Dig.Trp/Dig.Lys was 0.172 to 0.175 for female broilers, 0.163 to 0.170 for male broilers in starter stage; 0.167 to 0.175 for female broilers, 0.168 to 0.176 for male broilers in grower stage; 0.175 to 0.177 for female broilers in finisher stage. The optimal response Trp level in low-protein diets for growth performance can also positively regulate broilers’ immune system and stress status. The dynamic model of Dig.Trp/Dig.Lys daily requirement on average metabolic BW and ADG was *y* = 0.835 × BW^0.75^-1.745 × ADG-24.181 for females and *y* = −0.054 × BW^0.75^ + 0.434 × ADG-0.107 for males in starter phase; *y* = −0.208 × BW^0.75^ + 0.759 × ADG + 12.686 for females and *y* = −0.028 × BW^0.75^ + 0.523 × ADG-11.082 for males in grower phase; *y* = 0.393 × BW^0.75^-0.286 × ADG-91.961 for females and *y* = 0.146 × BW^0.75^ + 0.323 × ADG-55.300 for males in finisher phase. This research provides precise nutritional strategies to enhance poultry performance and efficiency.

## CRediT authorship contribution statement

**Yuying Zhang:** Writing – original draft, Project administration, Methodology, Investigation, Data curation. **Suxin Shi:** Validation, Project administration. **Gaoxiang Yuan:** Project administration. **Xiaoyi Li:** Project administration. **Zhouyang Gao:** Project administration. **Yongfei Hu:** Supervision, Methodology. **Yuming Guo:** Supervision, Methodology. **Dan Liu:** Writing – review & editing, Supervision, Methodology.

## Disclosures

None.
